# Neutralizing antibodies against SARS-CoV-2 of vaccinated healthcare workers in Taiwan

**DOI:** 10.1080/07853890.2024.2442533

**Published:** 2024-12-23

**Authors:** Seto Priyambodo, Kuang-Che Kuo, Ken-Pen Weng, Shih-Feng Liu, Guan-Da Syu, Ho-Chang Kuo

**Affiliations:** a Department of Biotechnology and Bioindustry Sciences, National Cheng Kung University, Tainan, Taiwan; b Department of Biochemistry, Faculty of Medicine, University of Mataram, Mataram, Indonesia; c Department of Pediatrics, Kawasaki Disease Center, Kaohsiung Chang Gung Memorial Hospital, Kaohsiung, Taiwan; d College of Medicine, Chang Gung University, Taoyuan, Taiwan; e Department of Pediatrics, Congenital Structural Heart Disease Center, Kaohsiung Veterans General Hospital, Kaohsiung, Taiwan; f School of Medicine, National Yang-Ming Chiao Tung University, Taipei, Taiwan; g Division of Pulmonary and Critical Care Medicine, Department of Internal Medicine, Kaohsiung Chang Gung Memorial Hospital, Kaohsiung, Taiwan; h Department of Respiratory Therapy, Kaohsiung Chang Gung Memorial Hospital, Kaohsiung, Taiwan; i International Center for Wound Repair and Regeneration, National Cheng Kung University, Tainan, Taiwan; j Medical Device Innovation Center, National Cheng Kung University, Tainan, Taiwan; k Center for Infection Control, National Cheng Kung University Hospital, College of Medicine, National Cheng Kung University, Tainan, Taiwan

**Keywords:** Taiwan healthcare workers, SARS-CoV-2 vaccination, AZD1222, mRNA-1273, neutralizing antibody

## Abstract

**Background:**

Vaccination is one of the best ways to control the SARS-CoV-2 outbreak. In Taiwan, healthcare workers were prioritized for vaccination, but the effectiveness of these vaccines for them remains unclear. Thus, it’s essential to examine their neutralizing antibodies after prime-boost vaccinations.

**Methods:**

In this prospective observational study, 514 healthcare workers from Chang Gung Memorial hospitals in Taiwan were included between 19 March 2021 and 21 August 2021. The two doses of COVID-19 vaccines were either a match or a mixing of AZD1222 and mRNA-1273, e.g. AZD1222 + AZD1222 (*n* = 406), mRNA-1273 + mRNA-1273 (*n* = 62), and AZD1222 + mRNA-1273 (*n* = 46). Blood specimens were drawn after two doses of vaccines, defined as post-vaccine days [median 34.00 days and interquartile range (IQR) 29.00–42.00 days], and examined for the neutralizing antibodies *via* SARS-CoV-2 neutralization kits. The results were analyzed as a percentage of inhibition based on the negative control.

**Results:**

After 2 vaccination doses, subjects with AZD1222 + mRNA-1273 (median 97.15%, IQR 96.06–98.06%) and mRNA-1273 + mRNA-1273 (median 97.47%, IQR 96.75–97.89%) exhibited higher neutralizing antibodies than those receiving AZD1222 + AZD1222 vaccines (median 71.28%, IQR 49.39–89.70%) (the percentage was referred to inhibition of surrogate virus). The post-vaccination days negatively impacted the neutralizing antibodies, except for the mRNA-1273 + mRNA-1273 group. The presence of fever, headache, and myalgia after the second dosage was reflected in the higher neutralizing antibodies (median of no fever 76.00% *vs.* fever 97.00%, *p* < 0.0001; median of no headache 76.00% *vs.* headache 95.00%, *p* < 0.0001; median of no myalgia 75.50% *vs.* myalgia 96.00%, *p* < 0.0001). The subjects with underlying diseases, including hypertension and cancer showed lower neutralizing antibodies (median of no hypertension 81.00% *vs.* hypertension 56.00%, *p* = 0.0029; median of no cancer 81.00% *vs.* cancer 56.00%, *p* = 0.0143).

**Conclusion:**

Heterologous prime-boost vaccines (AZD1222 + mRNA-1273) and two doses of mRNA vaccines are recommended. For future directions, we need to investigate the effectiveness of the vaccination against new SARS-CoV-2 variants.

## KEY MESSAGES

- Vaccination with AZD1222 + mRNA-1273 and mRNA-1273 + mRNA-1273 produced higher neutralizing antibodies than AZD1222 + AZD1222 in Taiwan healthcare workers.
- Neutralizing antibodies were associated with vaccine side effects, including fever, headache, and myalgia.
- Underlying diseases (hypertension and cancer) and time after vaccinations reduced neutralizing antibodies from the vaccinations.

## 1. Background

The novel Severe-Acute Respiratory Syndrome Coronavirus-2 (SARS-CoV-2) has impacted the world, causing severe illness or death to many people. Expanding vaccinations is considered one of the best ways to control the outbreak [1]. However, the emergence of these new vaccine developments presents a challenge globally, as many are introduced and administered to humans following national emergency authorization. COVID-19 vaccines, including Astra Zeneca (AZD1222 or ChAdOx1) and Moderna (mRNA-1273) vaccines, are widely accepted in Taiwan and internationally [2]. During their clinical trials under emergency authorization, the information about these vaccines is limited, especially in the Asian population.

In Taiwan, initial priority was given to vaccinating healthcare workers and social care workers, with subsequent rollout to clinically high-risk groups [3]. In the early stages in Taiwan due to the lack of vaccine supplies, a policy based on recommendations from the Joint Committee on Vaccination and Immunization suggested extending the prime-boost intervals to 12 weeks [4]. Also, the vaccination types were limited to AZD1222 and mRNA-1273. However, we do not know the immune responses to these vaccines in Taiwanese people. Since the healthcare workers play a crucial role in medical services in Taiwan, it is important to study their immune responses after different SARS-CoV-2 vaccines.

After immunization, the immune system will produce antibodies. Part of these antibodies, named neutralizing antibodies, can help to defend the cells from infections. Therefore, the neutralizing antibody levels are related to the level of protection [5,6]. Since the health workers are at the front line, it is necessary to evaluate the neutralizing antibodies after administering different types of SARS-CoV-2 vaccines. In this study, we examined the neutralizing antibody levels of serum samples from healthcare workers who received 2 doses of mixing or matching of Astra Zeneca (AZD1222) or Moderna (mRNA-1273) vaccine. We also investigated the factors that affect the neutralizing antibodies, e.g. age, underlying diseases, and vaccine-induced side effects.

## 2. Materials and methods

### 2.1. Subjects

In this prospective observational study, 792 healthcare workers from Chang Gung Memorial hospitals in Taiwan were recruited between 19 March 2021 and 21 August 2021. The inclusion criteria were in-house staff (non-students or staff in training) who had received two doses of the COVID-19 vaccine for adults and without previous infection. During the study period between 19 March 2021 and 21 August 2021, the accumulated infection rate of SARS-CoV-2 in Taiwan from 2019 was 0.06% of the total population. The subjects were divided into AZD1222 + AZD1222 (*n* = 406), mRNA-1273 + mRNA-1273 (*n* = 62), and AZD1222 + mRNA-1273 (*n* = 46). Within a week of vaccination, participants were requested to complete a survey reporting any adverse reactions they experienced. The exclusion criteria were missing medical records (*n* = 167), missing 2nd dose of vaccine (*n* = 28), or administrated other types of vaccines (*n* = 22). The minimum sample size was calculated using a sample size calculator from Raosoft with a 5% margin error, 95% confidence interval, and a normal distribution of 50% [7]. From the total population of 7411 healthcare workers in Chang Gung Memorial hospitals, the estimated minimum sample size was 366. Post-vaccine days were defined as the median and interquartile range (IQR) time between vaccination and the drawing of blood samples. The serums were collected after 2nd dose of vaccination and stored at −80 °C for further assays (median 34.00 days, IQR 29.00–42.00 days after vaccination/post-vaccine days). The serums were collected after 2nd dose of vaccination and stored at −80 °C for further assays (median 34.00 days, IQR 29.00–42.00 days after vaccination).

### 2.2. SARS-CoV-2 neutralizing antibody assays

The neutralizing antibodies in serums were determined by a SARS-CoV-2 surrogate virus neutralization kit according to the manufacturer’s protocols (GenScript, #A210506). Briefly, serums or controls were diluted 10-fold and mixed with RBD-HRP for 30 min at 37 °C. Then, 100 μl from the mixture was transferred to the 96 well-plates to perform competition against coated ACE2. After incubating for 15 min at 37 °C, the plates were washed, incubated with TMB solution, stopped solution, and immediately read the absorbance at a wavelength of 450 nm. The results were analyzed as a percentage of inhibition based on the negative control = (1 − (sample OD450/negative control OD450)) × 100%. According to the protocol, ≥30% was defined as positive neutralizing activity [8].

### 2.3. Statistical analysis

GraphPad Prism 10.1.2 was used for plots and statistical analysis. Antibody level, age, and post-vaccine days were expressed as median and IQR as they did not pass the normality test. For the continuous data, Kruskal-Wallis tests were used to determine significant differences among groups. The correlation between neutralizing antibodies and other parameters was assessed using Pearson linear correlations. The Mann-Whitney and Chi-square tests were used to compare categorized data. A *p*-value <0.05 was considered statistically significant.

## 3. Results

### 3.1. Characteristics of the healthcare workers

After we excluded the participants with missing clinical data, less than two doses of vaccines, or other vaccine types, we have 514 samples in this study. Among these healthcare workers, 406 individuals received homologous AZD1222 vaccines (labeled as AZD1222 + AZD1222), 46 individuals received heterologous AZD1222 vaccine followed by mRNA-1273 vaccine (labeled as AZD1222 + mRNA-1273), and 62 individuals received homologous mRNA-1273 vaccines (labeled as mRNA-1273 + mRNA-1273). The gender consisted of 45 males and 468 females, with a median age of 41.00 years (AZD1222 + AZD1222), 39.50 years (AZD1222 + mRNA-1273), and 38.00 years (mRNA-1273 + mRNA-1273). Among 514 subjects, 121 participants (23.50%) have underlying diseases, including hypertension, cancer, and diabetes (Table 1).

**Table 1.** Characteristics of the healthcare workers.

	AZD1222 + AZD1222, *n* = 406, (A)	AZD1222 + mRNA1273, *n* = 46, (B)	mRNA1273 + mRNA1273, *n* = 62, (C)	*p*-Value
Age [median (years), IQR]	41.00 (33.00–48.00)	39.50 (31.00–43.00)	38.00 (34.00–49.00)	A *vs.* B = 0.2743 A *vs.* C > 0.9999 B *vs.* C = 0.7152
Sex [*n* (male/female), %]	37/369 (10.03)	6/40 (15.00)	2/60 (3.33)	A *vs.* B = 0.389 A *vs.* C = 0.118 B *vs.* C = 0.054
Underlying disease (*n*, %)	98 (24.10)	10 (21.70)	13 (20.90)	A *vs.* B = 0.717 A *vs.* C = 0.5847 B *vs.* C = 0.922
Hypertension (*n*, %)	27 (6.60)	2 (4.30)	0 (0.00)	A *vs.* B = 0.5058 A *vs.* C = 0.0365* B *vs.* C = 0.1048
Cancer (*n*, %)	3 (0.70)	1 (2.10)	0 (0.00)	A *vs.* B = 0.3247 A *vs.* C = 0.4971 B *vs.* C = 0.2435
Diabetes mellitus (*n*, %)	11 (2.70)	1 (2.10)	4 (6.40)	A *vs.* B = 0.8305 A *vs.* C = 0.1192 B *vs.* C = 0.2955

The difference in age between groups was analyzed using the Kruskal-Wallis test. Data were expressed in median and IQR. The difference in gender and underlying diseases between groups were analyzed using the Chi-square test (*p < 0.05).

**Figure 1.**
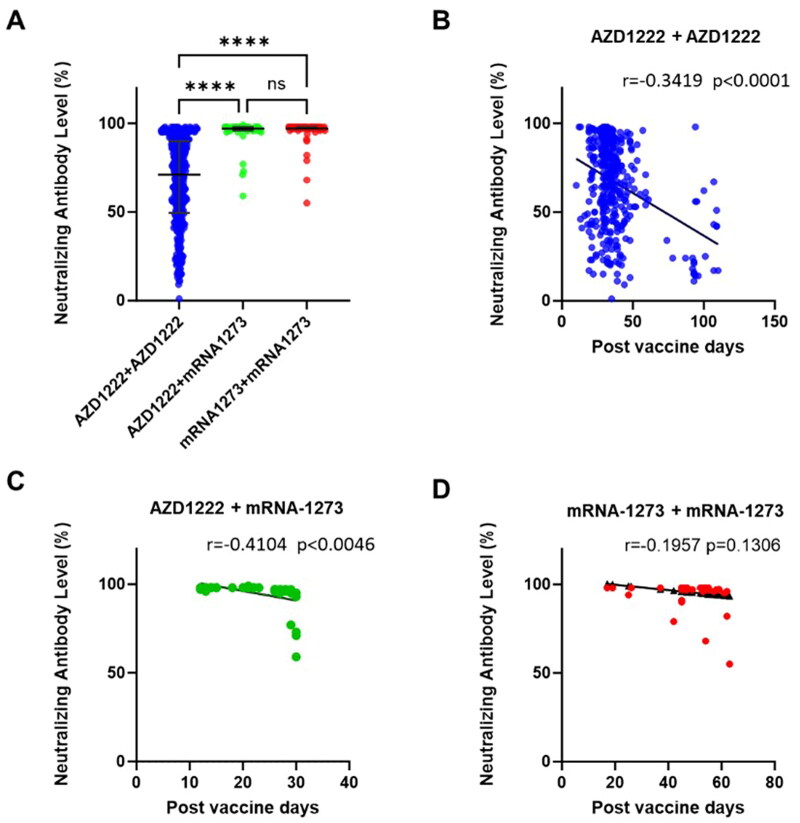
Neutralizing antibody levels between groups and post-vaccination days. Analysis of neutralizing antibody levels using the cPass SARS-CoV2 neutralizing antibody assay after 2 doses of vaccination. (A) Comparison of neutralizing antibody levels between 3 groups of participants who received the vaccine (1st and 2nd dose of vaccine combinations) from AstraZeneca (AZD1222) and Moderna (mRNA-1273) vaccines. (B) Longitudinal analysis of the correlation between neutralizing antibody levels and post-vaccination days who received the AZD1222 + AZD1222 vaccine group (blue color). (C). Longitudinal analysis of the correlation between neutralizing antibody levels and post-vaccination days who received the AZD1222 + mRNA-1273 vaccine group(green color). (D) Longitudinal analysis of the correlation between neutralizing antibody levels and post-vaccination days who received the mRNA-1273 + mRNA1273 vaccine group (red color). The difference in neutralizing antibody levels between groups was analyzed using the Kruskal-Wallis test (ns: not significant and ^****^p < 0.0001). Data were expressed as median and IQR. The Pearson correlation was used for the analysis of the correlation between neutralizing antibody levels and post-vaccination days.

The medians of neutralizing antibody levels were 71.28% (AZD1222 + AZD1222), 97.15% (AZD1222 + mRNA-1273), and 97.47% (mRNA-1273 + mRNA-1273) (%/percentage was referred to inhibition of surrogate virus). The participants from the AZD1222 + AZD1222 group showed lower neutralizing antibody levels compared to AZD1222 + mRNA-1273 and mRNA-1273 + mRNA-1273 group (*p* < 0.001) (as shown in Table 2 and Figure 1A). After the 2nd vaccination, the vaccine-induced adverse effects were the highest in AZD1222 + mRNA-1273 (93.50%), followed by mRNA-1273 + mRNA-1273 (72.60%), and the lowest in AZD1222 + AZD1222 (30.04%) groups (Table 2). We also analyzed qualitatively based on the manufacturer protocol, a.k.a., positive or negative neutralizing antibodies using the 30% as the cutoff suggested by the manufacturer. The results showed that the participants from the AZD1222 + AZD1222 group had significantly fewer positive participants (89.00%) compared to AZD1222 + mRNA-1273 (100.00%) and mRNA-1273 + mRNA-1273 (100.00%) (Figure S1).

**Table 2.** Neutralizing antibody levels, post-vaccine days, and adverse effects after the second vaccine.

	AZD1222 + AZD1222, *n* = 406, (A)	AZD1222 + mRNA1273, *n* = 46, (B)	mRNA1273 + mRNA1273, *n* = 62, (C)	*p*-Value
Neutralizing antibody level [median (%), IQR]	71.28 (49.39–89.70)	97.15 (96.06–98.06)	97.47 (96.75–97.89)	A *vs.* B < 0.0001^****^ A *vs.* C < 0.0001^****^ B *vs.* C > 0.9999
Post-vaccine days. (median, IQR)	34.00 (29.00–39.00)	26.00 (18.00–28.00)	53.00 (45.00–55.00)	A *vs.* B < 0.0001^****^ A *vs.* C < 0.0001^****^ B *vs.* C < 0.0001^****^
Second-dose adverse effect (*n*, %)	122 (30.50)	43 (93.50)	45 (72.60)	A *vs.* B < 0.0001^****^ A *vs.* C < 0.0001^****^ B *vs.* C = 0.0057**
Fever (*n*, %)	21 (5.30)	24 (52.17)	24 (38.71)	A *vs.* B < 0.0001^****^ A *vs.* C < 0.0001^****^ B *vs.* C = 1.392
Headache (*n*, %)	69 (16.99)	22 (47.82)	30 (48.38)	A *vs.* B < 0.0001^****^ A *vs.* C < 0.0001^****^ B *vs.* C = 0.9540
Myalgia (*n*, %)	58 (14.28)	31 (46.97)	33 (53.22)	A *vs.* B < 0.0001^****^ A *vs.* C < 0.0001^****^ B *vs.* C = 0.4793
Lymphadenopathy (*n*, %)	2 (0.40)	5 (10.86)	7 (11.20)	A *vs.* B < 0.0001^****^ A *vs.* C < 0.0001^****^ B *vs.* C = 0.9451
Chest pain (*n*, %)	8 (6.50)	0 (0.00)	6 (13.33)	A *vs.* B < 0.001*** A *vs.* C < 0.001*** B *vs.* C = 0.7057
Vomiting (*n*, %)	2 (0.49)	1 (2.10)	3 (4.80)	A *vs.* B = 0.1832 A *vs.* C = 0.0019** B *vs.* C = 0.4684
Diarrhea (*n*, %)	9 (7.31)	1 (2.32)	0 (0.00)	A *vs.* B = 0.3368 A *vs.* C = 0.8486 B *vs.* C = 0.3868
Skin rash (*n*, %)	7 (5.69)	1 (2.32)	2 (4.44)	A *vs.* B = 0.8264 A *vs.* C = 0.4226 B *vs.* C = 0.7422

The difference in neutralizing antibody levels and post-vaccination days between the groups was analyzed using the Kruskal-Wallis test. The difference in second dose adverse effect between groups was analyzed using the Chi-square test (**p < 0.01, ***p < 0.001, and ^****^p < 0.0001). Data were expressed in median and IQR.

### 3.2. Correlation of neutralizing antibodies, post-vaccination days, and participant age

It is known that the neutralizing antibodies would wane over time [9,10]. However, it is not well known in the different mixing or matching vaccines as well as the responses in healthcare workers in Taiwan. Correlation analysis of neutralizing antibody levels and post-vaccination days showed a negative association for AZD1222 + AZD1222 (*p* < 0.0001 and *r* = −0.3419) and mRNA-1273 + mRNA-1273 (*p* = 0.0046 and *r* = −0.4104) (Figures 1B,C). The correlation in the group of mRNA-1273 + mRNA-1273 was negligible, but it still showed a negative trend (*p* = 0.1306 and *r* = −0.1957) (Figure 1D).

**Figure 2.**
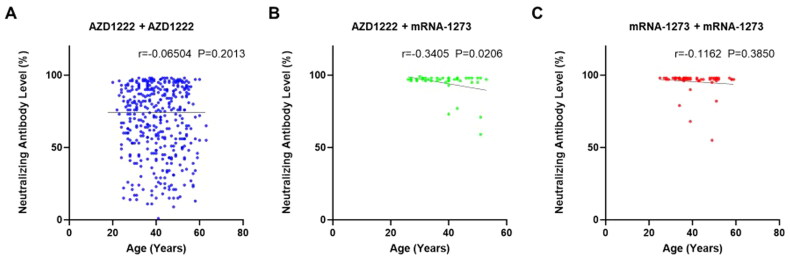
Neutralizing antibody levels and age. Correlation of antibody levels and age, measured using cPass SARS-CoV-2 neutralizing antibody assay after 2 doses of vaccination with age: (A) AZD1222 + AZD1222 vaccine group (blue color). (B) AZD1222 + mRNA-1273 vaccine group (green color). (C) mRNA-1273 + mRNA-1273 vaccine group (red color). The Pearson correlation was used for the analysis of the correlation between neutralizing antibody levels and age.

It is also known that the vaccine responses would decay with increasing age. Similarly, it is not well known in the different mixing or matching of COVID-19 vaccines and the responses in healthcare workers in Taiwan. Correlation analysis of neutralizing antibody level and age in the group of AZD1222 + mRNA-1273 showed a negative association with a weak correlation coefficient (*p* = 0.0206 and *r* = −0.3405) (Figure 2B). The association of neutralizing antibody level and age in the group of AZD1222 + AZD1222 and a group of mRNA-1273 + mRNA-1273 was insignificant, with a negative tendency (*p* = 0.2013 and *r* = −0.0950) and (*p* = 0.3850 and *r* = −0.1162), respectively (Figures 2A,C).

### 3.3. Neutralizing antibodies and vaccine-induced adverse effects

**Figure 3.**
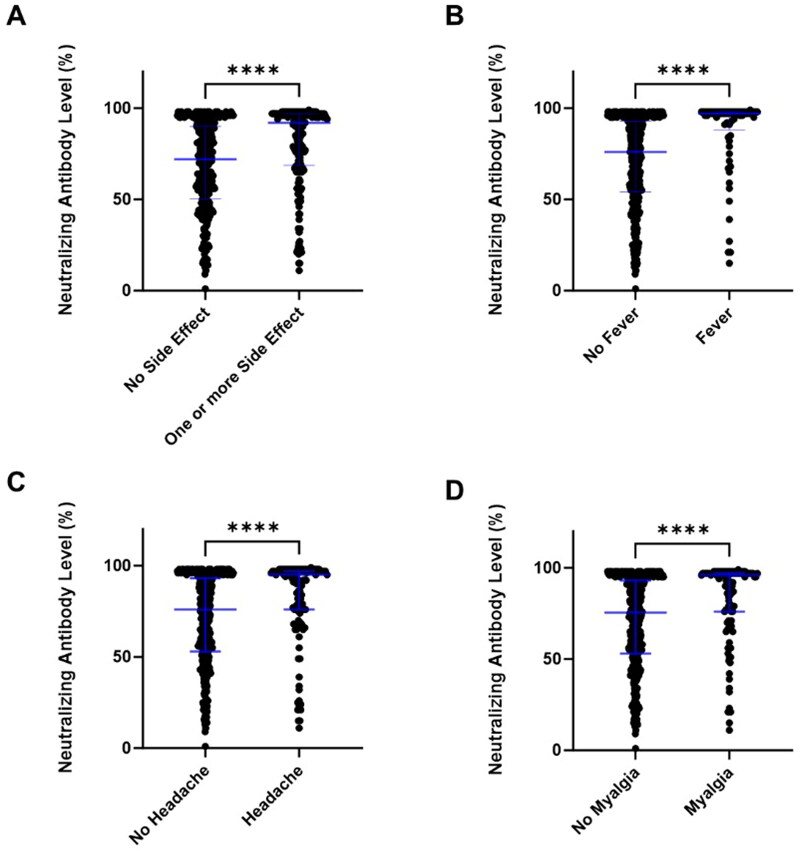
Neutralizing antibody levels and adverse effects after the second vaccine. (A) Comparison of neutralizing antibody levels in participants with and without adverse effects of 2nd dose vaccination in all participants. (B) Comparison of neutralizing antibody levels in participants with and without fever of 2nd dose vaccination. (C) Comparison of neutralizing antibody levels in participants with and without headache of 2nd dose vaccination. (D) Comparison of neutralizing antibody levels in participants with and without myalgia of 2nd dose vaccination. The Mann-Whitney test comparison analysis was used for the difference between adverse effects (^****^p < 0.0001). Data were expressed as median and IQR.

The vaccine-induced adverse effects or side effects are usually caused by the immune responses [11]. To investigate the mixing or matching vaccine episodes of healthcare workers in Taiwan, we followed up after 2nd dose of vaccination and recorded the adverse effects. Among vaccine-induced adverse effects in all three vaccine episodes, 92 participants (17.89%) had fever, 121 participants (23.50%) had headache, 122 participants (23.70%) had myalgia, 14 participants (2.72%) had lymphadenopathy, 14 participants (2.72%) had chest pain, 6 participants (1.16%) had vomiting, 10 participants (1.94%) had diarrhea, and 10 participants (1.91%) had skin rash. We further compared the neutralizing antibodies with or without adverse effects and found the higher neutralizing antibodies in subjects with one or more adverse effects after 2nd COVID-19 vaccine (*p* < 0.001) (Figure 3A). We also found that participants with fever, headache, and myalgia had higher levels of neutralizing antibodies (median of no fever 76.00% *vs.* fever 97.00% *p* < 0.0001; median of no headache 76.00% *vs.* headache 95.00% *p* < 0.0001; median of no myalgia 75.50% *vs.* myalgia 96.00% *p* < 0.0001) (Figures 3B–D). Other adverse effects, such as lymphadenopathy, chest pain, vomiting, diarrhea, and skin rash, did not affect the neutralizing antibody levels (data not shown).

### 3.4. Neutralizing antibodies and underlying diseases

**Figure 4.**
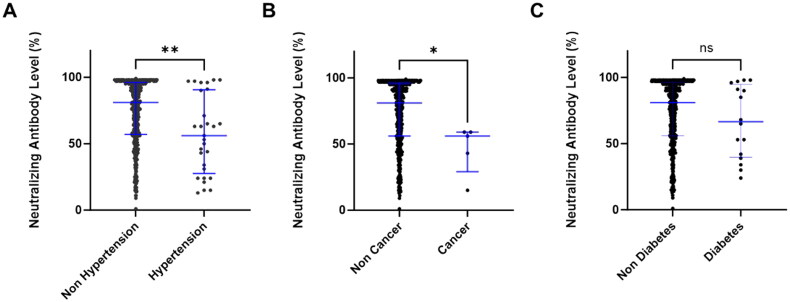
Neutralizing antibody levels and underlying diseases. Comparison of neutralizing antibody levels in all groups of vaccination according to participants with or without underlying diseases. (A) hypertension, (B) cancer, and (C) diabetes. The data was analyzed using the Mann-Whitney *U* test (ns: not significance, *p < 0.05, and **p < 0.01). Data were expressed as median and IQR.

The underlying diseases may affect the immune system as well as the COVID-19 vaccine responses [12]. We examined the medical records of 514 healthcare workers and identified that 29 participants (5.64%) had hypertension, 16 participants (3.11%) had diabetes, and 4 participants (0.77%) had cancer. We further analyzed the influence of underlying diseases in the neutralizing antibodies after 2nd COVID-19 vaccination. The results showed significant differences in the neutralizing antibodies in hypertension and cancer (median of no hypertension 81.00% *vs.* hypertension 56.00%, *p* = 0.0029; median of no cancer 81.00% *vs.* cancer 56.00%, *p* = 0.0143) (Figures 4A,B). On the contrary, the participants with diabetes showed the same antibody level compared to non-diabetes participants (Figure 4C).

## 4. Discussion

Our findings revealed that neutralizing antibody levels of serum samples from healthcare workers in Taiwan who received the AZD1222 + mRNA-1273 and mRNA-1273 + mRNA-1273 vaccines have higher levels than the AZD1222 + AZD1222 vaccine. This suggests that matching the AZD1222 + AZD1222 vaccine may not be sufficient to induce neutralizing antibody levels in all participants (Figure S1). Our findings align with previous reports on the immune response after two doses of ChAdOx1 nCov-19 (AZD1222) and heterologous prime-boost vaccines [13,14]. The neutralizing antibodies of a double dose of AZD1222 vaccines are lower compared to the combination of AZD1222 with the mRNA-1273 vaccines [15]. A study in Thailand with other mRNA vaccine boosters (Pfizer-BioNTech/BNT) also shows higher antibody levels than AZD1222 boosters in healthcare workers [16].

We observed that the neutralizing antibodies were similar between AZD1222 + mRNA-1273 and mRNA-1273 + mRNA-1273. Both combinations were enough to achieve high antibody levels against SARS-CoV-2 wild-type (Figure S1). Another report suggests that mRNA-1273 evokes higher immune responses against wild-type than AZD1222 [17]. In terms of multiple vaccinations, mRNA-1273 can further increase the antibody levels against spike protein after 2 doses of AZD1222 [18] [19]. Concerning the ongoing mutations, especially for the variants of concern (VOCs), it is important to know the immune responses after vaccination. Although this study did not analyze the immune responses against variants, our earlier study suggests mixing of AZD1222 and mRNA-1273 exhibits higher neutralizing antibodies against omicrons compared to the matching of AZD1222 or mRNA-1273 [20].

In general, the immune responses after vaccinations decline with age, especially in persons more than 50 years old [21] or more than 60 years old [15]. In our study, we found a downtrend (not significance) of neutralizing antibody levels in the age group (Figure S2). Perhaps due to the relatively small sample size, we only found a negative association between older age and antibody levels with a weak correlation in the group of AZD1222 + mRNA-1273. This suggests that older people still have the benefit of AZD1222 + mRNA-1273 vaccination [22,23]. The risks of adverse effects are higher in the second dose, such as pain at the injection site, fever, and paresthesia [24,25]. Fever, headache, and myalgia were the most inconvenient vaccine adverse effects experienced in the 2nd dose of vaccination in our research. We found that participants with fever, headache, and myalgia had higher levels of neutralizing antibodies (Figure 3). Others also indicate that IgG titers are associated with adverse outcomes after 2nd vaccination [26].

Underlying diseases can impact immune responses. Our results demonstrated that underlying diseases, such as hypertension and cancer, showed a negative impact on the neutralizing antibodies after vaccinations. Other studies found that neutralizing antibody levels were lowered in transplant patients, obese persons, smokers, and those with comorbidities [27,28]. However, these high-risk individuals will benefit from the additional booster vaccine doses [27]. Due to the limited sample size for multiple regression, this study did not analyze the impact of potential confounders on antibody responses.

## 5. Conclusion

Heterologous prime-boost vaccines (AZD1222 + mRNA-1273) and two doses of mRNA-1273 vaccines (mRNA-1273 + mRNA-1273) are recommended. Neu­tralizing antibody levels from healthcare workers in Taiwan who received the AZD1222 + mRNA-1273 and mRNA-1273 + mRNA-1273 vaccines have higher levels than the AZD1222 + AZD1222 vaccine. Neutralizing antibodies were higher in some side effects after vaccination, including fever, headache, and myalgia. Underlying diseases, such as hypertension and cancer, could reduce the neutralizing antibodies after vaccinations. For future directions, it is intriguing to investigate the effectiveness of vaccination against new SARS-CoV-2 variants as well as the waning immune responses of healthcare workers in Taiwan.

## Supplementary Material

Supplemental Material

## Data Availability

The datasets generated in this study are available from the corresponding author upon reasonable request (H.-C.K.erickuo48@yahoo.com.tw or G.-D.S.guanda@gs.ncku.edu.tw).
