# Supplementary material for: Neutralizing antibodies against SARS-CoV-2 of vaccinated healthcare workers in Taiwan
Source: Ann Med. 2024 Dec 23;57(1):2442533. doi: 10.1080/07853890.2024.2442533 (PMC11703416; doi:10.1080/07853890.2024.2442533)
Supplement: Supplemental Material [file IANN_A_2442533_SM9630.zip › Suppl_Mat/Supplementary figure caption.docx]

**Figure S1. Percentage of subjects with positive and negative neutralizing antibodies.** The percentage of positive and negative participants in each group (the participants with neutralizing antibody level >30% were counted as positive according to the cPass kit manufacturer). The ChiSquare test was used for analysis between groups. A vs B p = 0.0188 *, A vs C p = 0.0065 ** (*p < 0.05. ** < 0.01).

**Figure S2. Neutralizing antibody level stratified by age group.** The Neutralizing antibody level stratified by Age group of 21-35, 36-50 and 51-65 years. The Kruskal Wallis test was used for analysis between groups. Age 21-35 vs Age 51-65 p = ns, Age 36-50 vs Age 51-65 p = ns (ns: not significance). Data were expressed as median and IQR.
