# Supplementary figures and images for: Neutralizing antibodies against SARS-CoV-2 of vaccinated healthcare workers in Taiwan
Source: Ann Med. 2024 Dec 23;57(1):2442533. doi: 10.1080/07853890.2024.2442533 (PMC11703416; doi:10.1080/07853890.2024.2442533)

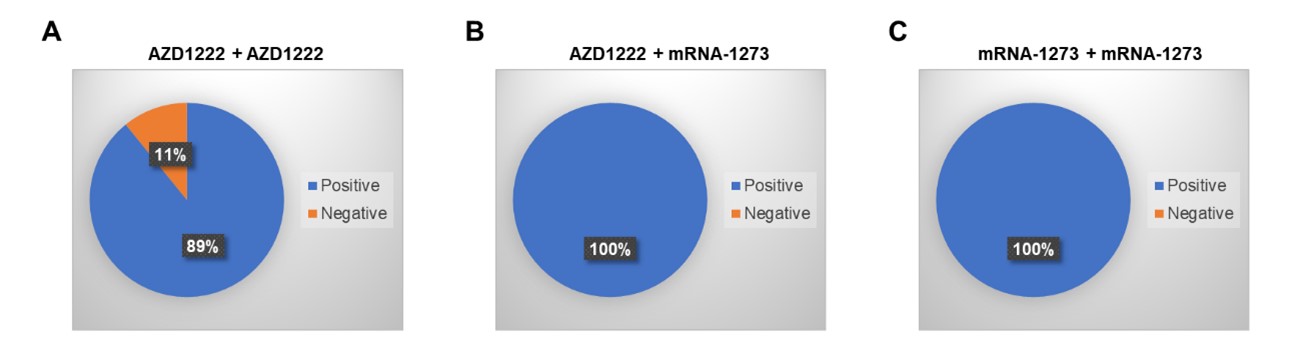

Supplement: Supplemental Material [file IANN_A_2442533_SM9630.zip › Suppl_Mat/Supplementary figure 1 S1.jpg]

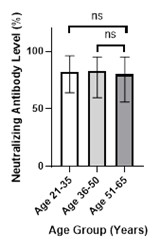

Supplement: Supplemental Material [file IANN_A_2442533_SM9630.zip › Suppl_Mat/Supplementary Figure 2 S2.jpg]
